# Dynamics of microbial community and enzyme activities during preparation of *Agaricus bisporus* compost substrate

**DOI:** 10.1038/s43705-022-00174-9

**Published:** 2022-09-23

**Authors:** Meghann Thai, Katarzyna Safianowicz, Tina L. Bell, Michael A. Kertesz

**Affiliations:** School of Life and Environmental Sciences, The University of Sydney, Sydney, NSW 2006 Australia

**Keywords:** Microbial ecology, Microbial ecology

## Abstract

Button mushrooms (*Agaricus bisporus*) are grown commercially on a specialized substrate that is usually prepared from wheat straw and poultry manure in a microbially-mediated composting process. The quality and yield of the mushroom crop depends critically on the quality of this composted substrate, but details of the microbial community responsible for compost production have only emerged recently. Here we report a detailed study of microbial succession during mushroom compost production (wetting, thermophilic, pasteurization/conditioning, spawn run). The wetting and thermophilic phases were characterized by a rapid succession of bacterial and fungal communities, with maximum diversity at the high heat stage. Pasteurization/conditioning selected for a more stable community dominated by the thermophilic actinomycete *Mycothermus thermophilus* and a range of bacterial taxa including *Pseudoxanthomonas taiwanensis* and other Proteobacteria. These taxa decreased during spawn run and may be acting as a direct source of nutrition for the proliferating *Agaricus* mycelium, which has previously been shown to use microbial biomass in the compost for growth. Comparison of bacterial communities at five geographically separated composting yards in south-eastern Australia revealed similarities in microbial succession during composting, although the dominant bacterial taxa varied among sites. This suggests that specific microbial taxa or combinations of taxa may provide useful biomarkers of compost quality and may be applied as predictive markers of mushroom crop yield and quality.

## Introduction

Button mushrooms, *Agaricus bisporus*, are one of the most widely cultivated edible mushrooms, with about 8 billion kg produced per year worldwide [1]. They are grown on a composted substrate that is traditionally made from wheat straw, stable bedding, poultry manure and gypsum. The ingredients used to make mushroom compost varies in different parts of the world; in Europe and the USA, for instance, there is a heavy dependence on stable bedding (horse manure) as the primary carbon and nitrogen source [2–4], in Australia almost no stable bedding is used, and in China rice straw is often used in place of wheat straw [5]. Smaller amounts of other agricultural by-products such as canola meal, soybean meal and cottonseed meal are often added to provide additional nitrogen and stimulate microbial activity at the start of composting, depending on seasonal availability.

**Fig. 1:**
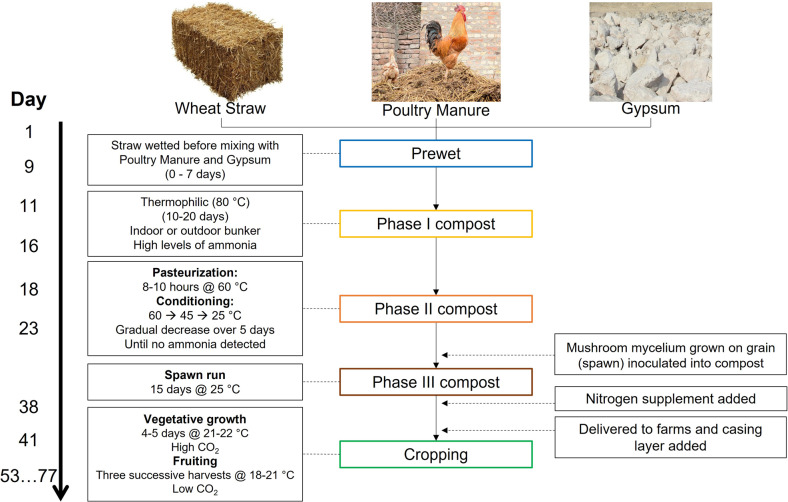
Summary of the mushroom composting process. Mushroom compost is produced from wheat straw, poultry manure and gypsum. The four phases of mushroom composting are indicated in the centre (Prewet, Phase I, Phase II, Phase III), followed by cropping. The key processes in each phase are shown on the left, together with the approximate number of days required for each phase. The timing and conditions given are typical of those observed in this study, but these may vary between compost yards.

Composting is a microbial process in which lignocellulosic waste materials are converted into a nutrient-rich humus-containing medium [6–8]. Details of the mushroom composting process vary between countries, but typically include a wetting phase to soften the straw raw materials and initiate straw breakdown, a thermophilic composting phase (Phase I, 70–80 °C) in which most of the structural components of the straw are degraded, and a pasteurization phase (Phase II, 60 °C) with subsequent conditioning at mesophilic temperatures (45 °C), in which the breakdown products are incorporated into microbial biomass and humic-lignin products in the final compost (Fig. 1). The *Agaricus* mycelium is introduced on a grain-based carrier (referred to as spawn) and allowed to proliferate throughout the compost. Mushroom production is then initiated by application of a low-nutrient layer of mixed peat and lime (referred to as casing), together with lowering the temperature and reducing CO_2_ levels in the growing rooms [9, 10]. Because the majority of the easily metabolizable plant metabolites are removed during the composting process and converted to microbial biomass and protein in the compost, the only organisms that can grow effectively on the finished compost are those that can access carbon either from the microbial biomass present or from residual lignin-humic complexes. This provides a nutritional environment that favors basidiomycetes over competing fungal pathogens (typically ascomycetes), yielding a compost that is highly selective for *Agaricus* under the cropping conditions used [11].

The main components of the cell wall of straw are structural carbohydrates, typically cellulose, hemicellulose (mainly xylan) and lignin, with pectin and related molecules providing structural cohesion. Wheat straw typically contains 40% cellulose, 25% hemicellulose, 23% lignin and 3% pectin [12], and these molecules provide the main growth substrate for the microbes present in the composting process. About 50% of the available xylan and cellulose is broken down during the thermophilic phases of composting [3], catalyzed by cellulases and xylanases that are released by the thermophilic bacteria and fungi in the compost. Lignin levels are largely unaffected, and much of the hemicellulose that remains at the end of the thermophilic phase is thought to be bound in lignin complexes. These hemicellulose fragments are released by the ligninase activity of *Agaricus* mycelium, but they are often highly substituted and are not well metabolized by *Agaricus* [13].

The succession of microbes that catalyze the composting process in a range of composting applications has been studied previously using traditional culture-based methodologies to isolate organisms on complex growth media (reviewed in Ryckeboer [14] and Kutzner [15]). Although a range of thermophilic fungi and bacteria was described, the cultivable bacteria reported from mushroom composts showed quite limited diversity, and were primarily related to *Bacillus* and to actinomycetes such as *Streptomyces* and *Thermoactinomyces* [14]. More recent work using molecular sequencing tools has revealed a much wider range of taxa [16–19]. In contrast to isolated bacteria, the thermophilic fungi isolated from mushroom compost were slightly more diverse [14]. These thermophilic fungi are essential in Phase II because they convert nutrients from the raw material into microbial biomass and in doing so contribute to the selectivity of mushroom composting. One particularly important thermophilic fungus, *Mycothermus thermophilus* (syn. *Scytalidium thermophilum*/*Humicola insolens*) aids the reassimilation of ammonia into the compost [2, 20, 21], and stimulates growth of the button mushroom mycelium. In the presence of *M. thermophilus*, hyphal elongation of *A. bisporus* doubles [20, 22] and fungal competitors of *A. bisporus*, such as *Chaetomium globosum*, are suppressed [2, 23]. *Mycothermus thermophilus* is the dominant fungal taxon in Phase II compost and makes up most of the microbial biomass in the compost [18, 22, 24–26], but it is just one player in a multifaceted microbial community.

Previous reports on bacterial and fungal succession in compost during mushroom composting have each studied individual compost yards, while suggesting significant variability in microbial diversity among different compost yards [2, 16, 17, 25–28]. In this study, we provide an in-depth analysis of fungal and bacterial diversity and succession at multiple time points throughout the mushroom composting process, and correlate this with activities of key compost enzymes. Variability among composting facilities was investigated directly by determining the bacterial community diversity in compost from five geographically separated composting yards in south-eastern Australia at three important timepoints in the composting process. This has allowed us to determine how bacterial community structure in mushroom compost varies among facilities, and whether variation can be explained by functional redundancy in species composition.

## Materials and methods

### Composting process

Compost for button mushroom cultivation was prepared by five commercial composting yards located in the east and southeast of Australia, referred to in this study as Yards A–E to preserve commercial confidentiality. Compost was prepared using the standard three-stage industrial composting process, with minor variations among yards (Table S1). Briefly, wheat straw was soaked for several days with recycled process water and was then mixed with gypsum, chicken manure, and any other additives used by specific yards. The blended raw materials were then subjected to an uncontrolled self-heating step, using underfloor aeration or frequent turning, with temperatures increasing to ~80 °C. The heating step typically lasted for 14–21 days with two to three turns (Phase I). Phase II composting (pasteurization and conditioning) was done either in bulk in a closed tunnel with floor aeration, or in trays, with pasteurization of the compost at 60 °C for 6–10 h followed by conditioning of the compost by gradual cooling to 45 °C over 4–5 days. The compost was further cooled to 25 °C before mixing in button mushroom spawn (*Agaricus bisporus* strain A15), together with a commercial supplement. The *Agaricus* mycelium was allowed to proliferate at 25 °C for 14 days (Phase III) in a closed tunnel (4 × 4 × 40 m). When required, spawned compost was overlaid with standard casing material (a mixture of peat, lime, and compost) and used for mushroom cropping in the Mushroom Research Unit at The University of Sydney. Three flushes of mushrooms were obtained under standard conditions.

### Compost sampling

For the first study, compost was sampled from Yard A in April–May 2012, with samples obtained from feedstocks and at 19 timepoints throughout the composting process over 39 days. Samples were collected at 3 to 4-day intervals throughout pre-wetting and Phase I, three times during Phase II and eight times during Phase III (spawn run) (Table 1). Five-fold replicate samples of ~50 g were collected randomly from different depths and heights within the stack by sampling during the regular compost turning process, as compost was moved between bunkers. Samples were stored in plastic zip-lock bags and immediately frozen at −20 °C. Frozen samples were ground in liquid nitrogen and kept at −80 °C until used.

**Table 1:** Sampling day, and enzyme activities in selected compost samples.

Process day	Description	Composting phase	Amylopectinase	ß-glucosidase	Cellulase	Chitinase	FDA hydrolysis	Invertase	Peroxidase	Protease	Xylanase
			nmol glucose min^−1^ g^−1^	nmol MUF min^−1^ g^−1^	nmol glucose/min/g	nmol MUF min^−1^ g^−1^	nmol fluorescein min^−1^ g^−1^ g	nmol glucose min^−1^ g^−1^	Absorbance @ 450 nm min^−1^ g^−1^	nmol AMC/min/g	nmol glucose min^−1^ g^−1^
0	Supplement	Feedstocks	11	bdl	1.7	bdl	bdl	bdl	bdl	17.3	bdl
0	Cotton hulls	Feedstocks	949	bdl	30.8	bdl	bdl	0.7	bdl	49.4	bdl
0	Chicken manure	Feedstocks	1450	bdl	11.8	bdl	bdl	4.7	3.7	110.0	192.2
0	Gypsum	Feedstocks	59	bdl	4.0	bdl	bdl	bdl	1.1	5.2	bdl
0	Feather meal	Feedstocks	26	bdl	bdl	bdl	bdl	0.4	bdl	0.8	bdl
0	Brew	Feedstocks	6387	bdl	50.8	51.8	bdl	25.6	7.2	91.4	57.5
1	Bale setup	Pre-wet	1521 ± 1518	47.9 ± 5.7	120.8 ± 61.5	7.0 ± 2.3	40.2 ± 12.3	39.6 ± 10.6	bdl	131.4 ± 10.6	365.6 ± 103.2
2	24 h of wetting	Pre-wet	604 ± 41	47.8 ± 10.1	42.2 ± 25.1	9.4 ± 2.8	43.9 ± 9.3	47.6 ± 9.1	3.9 ± 1.4	121.7 ± 25.4	165.2 ± 6.5
4	72 h of wetting	Pre-wet	239 ± 176	13.5 ± 5.9	24.0 ± 3.5	9.6 ± 3.7	73.0 ± 19.4	7.2 ± 1.7	1.3 ± 1.5	83.8 ± 8.6	44.7 ± 14.0
7	Rick turn start	Pre-wet	411 ± 184	5.0 ± 2.3	15.2 ± 9.0	4.5 ± 1.0	53.3 ± 12.8	2.4 ± 2.4	0.1 ± 0.7	53.1 ± 7.8	31.1 ± 34.9
9	Rick turn end	Pre-wet	bdl	0	2.9 ± 2.8	0.3 ± 0.2	1.3 ± 2.4	0.4 ± 0.3	bdl	14.4 ± 4.1	bdl
11	Phase I tunnel 2	Phase I	41 ± 13	11.0 ± 3.9	8.1 ± 1.7	3.7 ± 0.2	21.2 ± 5.0	0.2 ± 0.1	8.1 ± 1.9	36.7 ± 5.3	62.5 ± 20.7
13	Blend 1	Phase I	46 ± 12	3.4 ± 1.3	3.7 ± 1.5	0.1 ± 0.2	1.0 ± 2.9	0.2 ± 0.1	bdl	12.3 ± 3.1	17.8 ± 10.9
16	Blend 2	Phase I	bdl	6.1 ± 1.8	4.0 ± 1.1	1.1 ± 0.3	7.9 ± 2.1	0	bdl	8.5 ± 1.8	bdl
18	Pasteurization	Phase II	109 ± 34	10.2 ± 2.3	6.6 ± 3.9	1.8 ± 0.3	16.1 ± 2.3	0.3 ± 0.2	0.9 ± 0.5	67.8 ± 5.0	18.2 ± 4.1
20	Conditioning	Phase II	275 ± 61	20.0 ± 3.3	26.4 ± 4.1	1.0 ± 0.1	20.8 ± 6.1	0.6	1.6 ± 1.2	47.4 ± 2.7	249.8 ± 19.2
23	Conditioning end	Phase II	264 ± 104	32.1 ± 3.1	39.0 ± 10.4	1.5 ± 0.4	32.7 ± 12.6	1.1 ± 0.7	1.6 ± 1.3	71.8 ± 16.2	359.0 ± 37.6
25	Spawn run 1	Spawn run	586 ± 57	32.9 ± 8.3	38.0 ± 3.4	3.7 ± 0.6	41.5 ± 8.8	2.0 ± 0.3	2.7 ± 0.6	73.5 ± 14.9	206.4 ± 34.0
27	Spawn run 2	Spawn run	907 ± 233	24.0 ± 5.9	62.1 ± 5.3	1.5 ± 0.4	45.9 ± 14.4	2.9 ± 1.1	0.8 ± 1.3	54.0 ± 13.4	352.0 ± 55.6
29	Spawn run 3	Spawn run	386 ± 53	23.4 ± 5.6	32.8 ± 8.0	2.5 ± 0.2	90.9 ± 20.9	1.6 ± 0.4	5.8 ± 1.3	53.7 ± 13.7	219.1 ± 39.8
31	Spawn run 4	Spawn run	539 ± 97	4.1 ± 1.0	29.0 ± 8.3	bdl	12.1 ± 1.2	3.2 ± 1.0	19.5 ± 5.2	34.6 ± 6.1	296.0 ± 92.9
33	Spawn run 5	Spawn run	323 ± 65	5.8 ± 0.3	39.4 ± 7.7	3.6 ± 0.5	27.6 ± 6.9	3.1 ± 0.4	20.6 ± 9.3	28.6 ± 7.0	158.8 ± 50.3
35	Spawn run 6	Spawn run	605 ± 48	3.1 ± 0.9	9.1 ± 3.5	bdl	2.8 ± 2.1	1.1 ± 0.3	46.9 ± 4.9	11.1 ± 0.4	129.8 ± 44.7
37	Spawn run 7	Spawn run	57.8 ± 1	1.6 ± 0.3	2.4 ± 0.9	2.0 ± 0.2	17.5 ± 4.0	0	43.8 ± 7.1	8.8 ± 2.2	24.5 ± 10.4
39	At casing	Spawn run	118 ± 40	bdl	bdl	bdl	bdl	0.3 ± 0.2	71.3 ± 1.0	3.7 ± 1.1	23.0 ± 5.8

Methods used to measure enzyme activities are outlined in Supplementary Table S1^a^.

^a^FDA fluorescein diacetate, MUF methylumbelliferone, AMC 7-amino-4-methylcoumarin, bdl below detection limit.

Comparative data from multiple composting facilities were obtained in a second study, for which compost was sampled from Yards A–E in July–August 2017. Single samples of ~500 g were collected randomly at the end of Phase I, between pasteurization and conditioning in Phase II (where technically possible), and at the end of Phase II. Single samples were taken by hand from the face of the compost pile, stored in plastic zip-lock bags and transported to the laboratory within 1–3 days. Each bulk sample was mixed thoroughly, and five subsamples were taken and stored at −20 °C.

### Compost physicochemical measurements

Water content of the compost was measured gravimetrically by weighing subsamples of compost before and after oven drying at 105 °C for 24–48 h. Moisture content was expressed as a percentage of the fresh weight. Ash content was determined gravimetrically after heating the dried sample for 2 h in a muffle furnace at 550 °C. pH and electrical conductivity of casing and compost extracts were determined using a pH meter (pH Cube, TPS, Queensland, Australia) and digital conductivity meter (Model PTI-18, Activon Scientific Products Co, New South Wales, Australia) in 1:10 water extracts (180 rpm shaking, 1 h, room temperature). The tubes were held at room temperature for 2 h to allow particulates to settle before measurements were taken.

Total carbon (C), nitrogen (N), and sulfur (S) content of dried and finely ground samples of feedstocks and compost substrates were determined by combustion (Vario Max CNS, Elementar Analysensysteme GmbH, Hanau, Germany). Total water-extractable C and N was measured using a TOC-analyzer (TOC-V CSH, TNM-1, Shimadzu, Kyoto, Japan). Frozen samples (1.2 g) were extracted with 0.05 M K_2_SO_4_ (25 ml) at room temperature for 1 h with shaking (200 rpm). Extracts were filtered through filter paper (Whatman Grade 42), and total soluble C and N determined following the manufacturer’s instructions. Sodium phthalate and KNO_3_ solutions were used as C and N standards.

### Enzyme assays

Activity of nine enzymes in water extracts of compost samples was assayed using published colorimetric and fluorometric methods (Table S2), modified as needed to fit 96-well format. Compost extracts were prepared at room temperature by suspending 0.65 g of ground, frozen sample in 6 ml of sterile ultrapure water in 15 ml polypropylene tubes and shaking on an orbital shaker (180 rpm) for 30 min. Particulates were removed by centrifugation at 1500 × *g* (Falcon 6/300, MSE) and the clarified supernatants were stored on ice for up to 4–5 h until used.

Enzyme activity measurements were made using a plate spectrophotometer (BioTek Synergy H1, Agilent, California, USA) at times optimized to capture the amounts of product in the linear phase of enzyme activity. Enzymatic activity was expressed as μg of product generated per g of dry material per h.

### DNA extraction, amplification, and sequencing

For the first study, total compost DNA was extracted using a method adapted from Yeates and Gillings [29] Ground compost samples (0.3 g) were suspended in lysis buffer (6X; 1% (w/v) SDS, 1% (w/v) polyvinylpolypyrrolidone, 60 mM EDTA pH 8.0, 300 mM Tris-HCl pH 8.0), and lysed using a homogenizer (MoBio Laboratories Inc., California, USA) at 2000 rpm for 5 min. After protein precipitation with 1.2 M potassium acetate, DNA was recovered with a glass milk Binding matrix solution (MP Biomedicals, California, USA), diluted 1:6 with 6 M guanidine isothiocyanate. Bacterial diversity was analyzed using primers 515F and 806R [30] to amplify the V4 16S rRNA gene hypervariable region, and fungal diversity was measured with primers amplifying the ITS2 region (ITS3F and ITS4R) [31]. Paired-end Illumina sequencing was done using the Illumina MiSeq platform at University of Boulder (Colorado) and at RTL Genomics (Lubbock, Texas, USA).

Quantitative PCR was done with a CFX96 Touch Real-Time System (Bio-Rad, California, USA), using the 16S and ITS primer pairs described above. Purified amplicon standards for quantification were generated from compost DNA.

For the second study, total compost DNA was extracted according to Lever [32] with some modifications. Ground compost samples (200 mg) were suspended in 200 mM sodium hexametaphosphate (100 μl), lysis buffer 1 was added (30 mM Tris/HCl, 30 mM EDTA, 800 mM guanidinium chloride, 0.5% (v/v) Triton X-100, pH 10.0) (500 μl), and the samples were lysed using a homogenizer (MoBio Laboratories Inc.,) at 2000 rpm for 5 min. Lysis buffer 2 (2.5 M sodium chloride, 2% (w/v) cetyltrimethylammonium bromide, 0.1% (w/v) polyvinylpolypyrrolidone) (500 µl) was added, followed by incubation at 65 °C with agitation (500 rpm) for 30 min and centrifugation, Supernatants were extracted once with an equal volume of chloroform:isoamyl alcohol (24:1) and DNA was recovered from the aqueous phase using DNA binding magnetic beads (GE Life Sciences, Australia) in SPRI solution, following the manufacturer’s instructions. Bacterial diversity was analyzed using primers 341F and 806R [33, 34] to amplify the V3-V4 16S rRNA gene hypervariable region, with the Illumina MiSeq platform (paired 300 bp read lengths) at the Australian Genome Research Facility (Melbourne, Australia).

Sequencing data for both studies are available at NCBI SRA under BioProject PRJNA867030.

### Bioinformatics

Raw FASTQ files were processed in R v3.6.1 [35]. Raw read quality was determined using FastQC. Trimming and filtering was determined using the DADA2 function “filterAndTrim” [36], discarding forward and reverse reads with an expected error score higher than 3 and 4, respectively. Low quality reads were removed during trimming and filtering by setting “truncLen” parameters to 285 and 240 for the forward and reverse reads, respectively. Forward and reverse primers were trimmed from the 5’ end by setting the “trimLeft” function to 17 and 20, respectively. The sequences were denoised and dereplicated using the “dada” and “derep” functions, unique sequences were merged with a minimum overlap of 20 base pairs and a sequence table was constructed with the resulting sequence variants. Rarefaction curves are shown in Fig. S1.

Taxonomy was assigned using a pre-trained SILVA Naïve Bayes classifier clustered at 99% identity (SILVA release v132) [37]. Species assignment was done in a separate step using the SILVA release v132 for species assignment. 16S gene sequences that were affiliated with chloroplasts and mitochondria were removed prior to downstream analysis. Sequence variants which occurred in fewer than three samples and with fewer than three reads in each of these samples were also removed (singletons and doubletons). A phylogenetic tree was constructed using the packages “phangorn” [38] and “DECIPHER” [39], using the neighbor-joining method.

Statistical analysis was done using the packages “phyloseq” [40] and “vegan” [41]. All graphs and plots were visualized using “ggplot2” [42]. Shannon and Simpson alpha-diversity analyses were performed using the “plot_richness” function from the phyloseq package before singletons and doubletons were removed from the dataset. Differences in the bacterial community (beta-diversity) were analyzed in R [35] using a canonical analysis of principal coordinates with unweighted UniFrac as the distance metric.

## Results

Compost samples for the initial study were taken from 19 different timepoints during the pre-wet phase (bale-wetting and windrowing), Phase I (thermophilic), Phase II (pasteurization/conditioning), and Phase III (*Agaricus* spawn run) of a standard industrial composting run. The total yield of *Agaricus bisporus* obtained from the studied compost was 61.3 kg m^−2^ (460 g kg^−1^ compost), in four fruiting flushes (yielding 23.2, 15.6, 8.8 and 3.4 kg m^−2^ of mushroom caps, respectively). These yields are comparable or higher than standard yields obtained in the Australian mushroom industry, confirming the high productivity and representative nature of the composting run studied.

### Physicochemical changes during the composting process

The initial pH of the compost mix after addition of gypsum to the straw blend was 8.0, and it remained at this level during the pre-wet phase and Phase I, but decreased during pasteurization, conditioning and spawn run to a final value of pH 6.3. The electrical conductivity of the compost was stable at 2.5–3.0 μS throughout (Fig. S1).

The total C content of the compost decreased slowly from 48% (w/w) at bale break to 41% (w/w) when casing was applied at the start of the production phase, due to the loss of C through microbial respiration during composting. The ash content increased correspondingly from 7% (w/w) to 26% (w/w). Interestingly, total N and S content of the compost also increased slowly during the composting process to final values of 2.6% (w/w) for N and 2.2% (w/w) for S, but this increase probably just represents the retention of N and S despite overall loss of C. Extractable C and N levels increased briefly during the pre-wet phase, but then decreased again to base levels of 20 mg g dw^−1^ for carbon and 2–3 mg g dw^−1^ of N (Fig. S2). The moisture content of the compost increased during the initial bale wetting stage to 70% (w/w) and was maintained at 70–80% during Phase I, decreasing to 60–70% in Phase II and during spawn run (Fig. S3).

### Extracellular enzyme activities during composting

Conversion of the macromolecular components of compost feedstocks, especially proteins and the structural compounds cellulose, hemicellulose, and lignin, is catalyzed primarily by extracellular enzymes secreted by mesophilic and thermophilic bacteria and fungi. Key changes in the enzymatic profile occurred in the pre-wet phase, during the thermophilic phase, and at the end of spawn run. During initial bale wetting and pre-wet stage (process days 1–5), high activities were observed for most of the enzymes tested (Table 1). The high activities of invertase, amylopectinase and protease enable mesophilic compost organisms rapidly to utilize the soluble sugars, starch and protein made available in the substrate through the wetting process, and these activities decreased to low levels by day 9. Invertase and amylopectinase were not substantially active later in the composting process, possibly in response to depletion of their substrates. However, the enzymes responsible for degradation of cellulose (cellulase, β-glucosidase) and hemicellulose (xylanase) were also highly active at the start of the process and decreased in activity during the pre-wet period. These enzyme activities are therefore likely to be affiliated with mesophilic organisms that initially benefited from increased nutrient availability, but then decreased in activity with increasing temperature in the windrows. This is consistent with the transient increase seen in overall microbial activity (FDA hydrolysis) by day 5, with a subsequent reduction in activity by day 9.

Phase I was a highly aerobic phase, with air actively blown through the compost in large and enclosed tunnels. There was an immediate but transient increase in activity of cellulase/β-glucosidase, xylanase, protease, chitinase and overall microbial activity at the start of Phase I representing rapid growth of thermophilic organisms under these conditions. The transient pulse in enzyme activities was followed by a slow increase in activity of all these enzymes throughout the rest of Phase I, through Phase II (pasteurization and conditioning), and into the spawn run. Protease and β-glucosidase activities were especially high during the conditioning process, as the compost cooled after pasteurization, but the most notable aspect of this period of the process was the profile of xylanase and cellulase activities during the spawn run. Activity of these two enzymes increased to a maximum around day 25 and decreased again as colonization of the compost by *Agaricus* was completed, consistent with displacement of thermophilic cellulose-degrading fungi (especially *Mycothermus*) by the growing *Agaricus* mycelium.

Efficient colonization of the compost by *Agaricus* led to an increase in total microbial activity (FDA hydrolysis) but was also reflected in increasing peroxidase activity from day 30 onwards, corresponding with the onset of *Agaricus-*mediated lignin degradation. Chitinase activity also increased at the end of spawn run, suggesting enhanced activity of organisms producing cell wall degrading enzymes targeting *Agaricus* mycelium.

### Microbial populations in mushroom compost

**Fig. 2:**
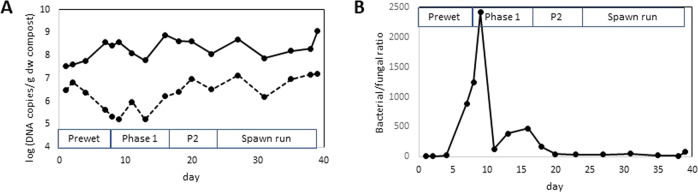
Total bacterial and fungal populations in compost at selected timepoints during composting. **A** Bacterial population—solid line; fungal population—dotted line, **B** bacterial/fungal ratio. Microbial populations were measured by qPCR using universal primers 515F and 806R for bacterial populations, and ITS3F and ITS4R for fungal populations.

The total population of bacteria in compost (measured as 16S rRNA gene copies per g dry weight of compost) was significantly higher than the fungal population (ITS copies per g dry weight of compost) throughout the composting process (Fig. 2). The size of the fungal population decreased during the pre-wet phase, while bacteria proliferated under these conditions, and the bacteria:fungi ratio reached almost 2500 at day 9. This ratio decreased sharply as temperature increased during Phase I, and mesophilic bacteria were eliminated. The fungal population grew steadily during Phase I and pasteurization/conditioning as thermophilic species proliferated. Interestingly, no substantial increase in fungal numbers was observed during spawn run, consistent with the concept that *Agaricus* obtains most of its nutrition through degradation of the biomass of other fungi such as the thermophilic *Mycothermus*. The bacterial population increased 10-fold during Phase I but decreased again during pasteurization and conditioning and there was always 10–50 times more bacteria than fungi. The size of the bacterial population increased together with *Agaricus* during spawn run, suggesting that the bacteria may have colonized the *Agaricus* hyphae, or otherwise benefited from the presence of this organism.

### Succession of bacterial communities in mushroom compost

Analysis of bacterial diversity in mushroom compost at 19 timepoints during the composting process revealed a total of 3240 different OTUs, with up to 1134 different OTUs present in any given sample (Table S4). Bacterial diversity increased slowly during the pre-wet phase, reaching a maximum at the end of Phase I. Removal of mesophilic organisms during pasteurization led to a steep fall in bacterial diversity; this decrease persisted through the conditioning period and during growth of the *Agaricus* mycelium, suggesting that these conditions were selective for a specific group of organisms.

**Fig. 3:**
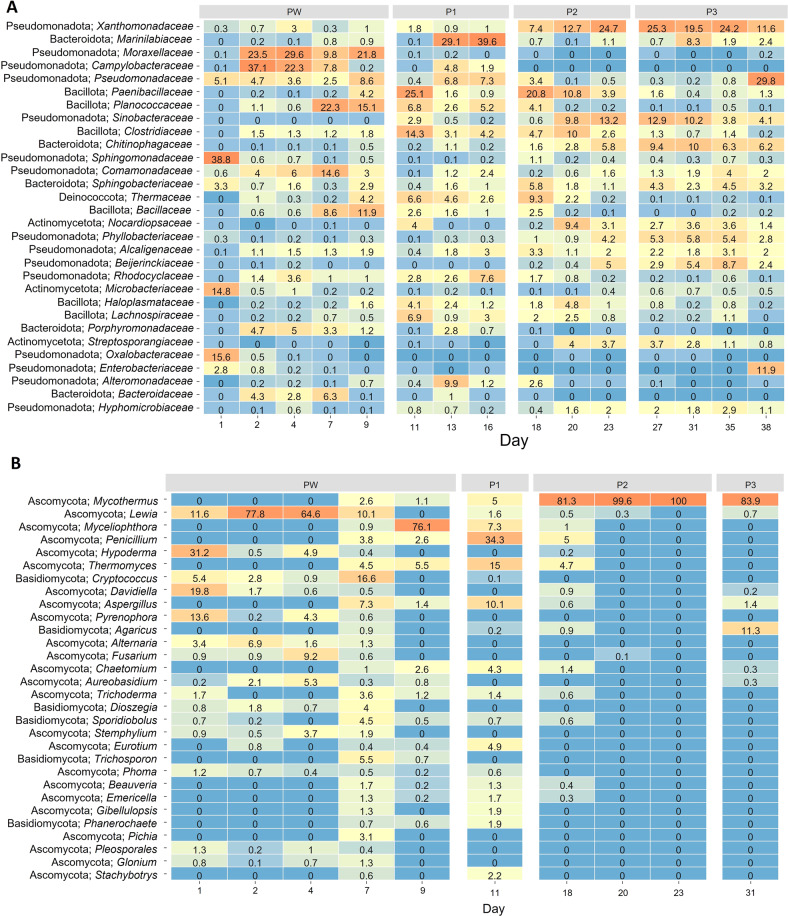
Relative abundance of microbial taxa in mushroom compost at selected timepoints. **A** Bacterial diversity (phylum/family). **B** Fungal diversity (phylum/genus). Relative abundances of the 30 most abundant taxa were center log ratio transformed and are displayed as colors ranging from blue (low) to red (high). Numbers indicate relative abundance (%) within each sample. PW prewet, P1, P2, P3 phases 1–3.

The dynamics of specific bacterial taxa during production of mushroom compost can be delineated into three main periods (Fig. 3). During the pre-wet phase and early Phase I, the dominant bacteria present changed frequently, with populations growing quickly, and then disappearing equally rapidly as they were overgrown by other species. *Arcobacter* made up nearly 30% of the total bacterial population at the start of composting, possibly derived from the poultry manure or from the recycled process water used for straw wetting. Several strains of *Acinetobacter* were also dominant (20–30% of overall population) during the early pre-wet phase, while the subsequent dominant taxa were *Solibacillus* and *Comamonas*, followed by *Pseudomonas* and *Bacillus*, largely mesophilic organisms which are characteristic of the straw and field origin of the raw materials. As Phase I progressed, the dominant taxa were *Bacillus, Paenibacillus* and uncharacterized Clostridia and Proteobacteria, followed by *Ruminofilibacter*, which made up to 20% of bacteria present in mid-Phase I. The frequent succession in dominant organisms presumably reflected the depletion of preferred C sources for each species from the compost, and the rapidly changing environmental conditions as the temperature of the compost increased.

The second distinctive period in the composting process was the end of Phase I/start of Phase II. At this point, species evenness increased, and individual organisms became less dominant, reflecting the increase in diversity seen at the end of Phase I. *Thermus* made up 6% of the bacterial community together with a thermophilic *Sphingobacterium* and a species of *Luteimonas*.

The bacterial community changed completely after pasteurization with the rapid variation seen earlier in composting replaced by comparative stability. The dominant organism throughout conditioning and spawn run was the heterotrophic nitrifier, *Pseudoxanthomonas taiwanensis*. The population size of this species increased quickly after pasteurization, and it made up about 15% of the bacterial compost community from this point until almost the end of the spawn run.

### Succession of fungal communities in mushroom compost

Fungal community dynamics during composting followed a similar pattern to that found for bacteria, although the overall fungal diversity was much lower (only 340 OTUs found with ITS3-ITS4 amplification) (Table S5). In addition, it was not possible to measure the diversity of compost fungi accurately in later samples than mid-spawn run, because *Agaricus* dominated the amplicon obtained from these timepoints. As with bacteria, the early stages of composting were characterized by a rapid succession of different taxa, responding to changing nutrient availability and temperature conditions. Although the wheat straw feedstock carried a range of different fungi (mainly in the families *Pleosporaceae, Rhytismataceae* and *Davidiellaceae*), the compost in pre-wet was dominated by the pleospore, *Lewia infectoria*, and gradually replaced by an uncharacterized ascomycete and *Myceliophthora* (Fig. 3). During Phase I there was a transient increase in *Thermomyces*, followed by rapid colonization of the compost by the thermophilic fungus, *Mycothermus thermophilus*, which made up more than 80% of the fungal population in the compost from mid-Phase II until it was overgrown by *Agaricus* in mid-spawn run.

### Microbial diversity variability among compost yards

All the composting yards sampled used the same fundamental composting process and the same main raw materials (poultry manure, wheat straw and gypsum), but there were differences among them in scale and process details (Table S1). Phase I compost production varied from 80 to 1600 t per crop, and three of the yards provided additional N either as inorganic supplements (urea or ammonium sulfate) or organic materials (e.g., cottonseed meal or soybean meal). The length of the pre-wetting period varied (2–14 days), as did the length of Phase I (9–21 days). The Phase II process, by contrast, was relatively similar at all yards.

**Fig. 4:**
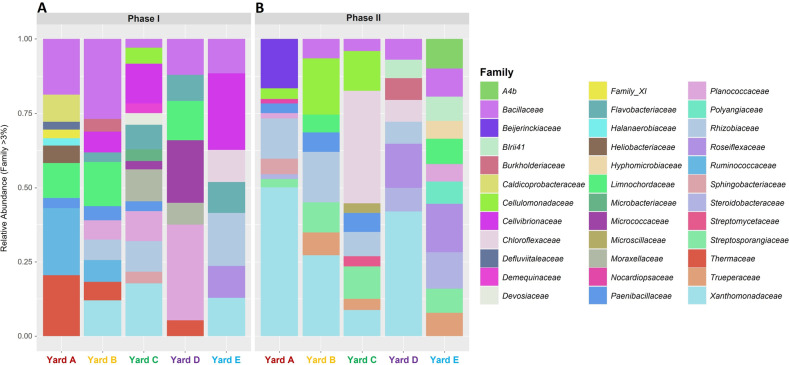
Relative abundance of bacterial taxa from five geographically distinct compost yards. **A** End Phase I, **B** end Phase II. Rare taxa with a relative abundance of <3% and taxa that were not classified to family are not shown.

To determine the effect of this variation across compost yards, bacterial community composition was measured in end-Phase I and end-Phase II compost at five yards from four Australian states (Fig. 4). At the end of Phase I, *Bacillaceae* was the only taxon consistently present in all compost yards with considerable differences in all other taxa. Yards A and B revealed similar bacterial communities, with a high proportion of *Ruminicoccaceae* and *Limnochordaceae*, and thermophilic bacteria in the *Thermaceae* and *Bacillaceae* families (15–30%). Yard A had a much higher proportion of *Thermus* than Yard B, possibly because it is a larger enterprise, allowing a high temperature to be more easily maintained throughout the compost pile (Table S1). In Yards B, C and E, common families were *Cellvibrionaceae*, *Xanthomonodaceae* and *Flavobacteriaceae* (2–10%), while the bacterial profile for Yard D had a higher proportion of mesophilic taxa such as *Planococcaceae* and *Micrococcaceae* (~7–12%). This may be because Phase I was done outdoors and, as a consequence, lower temperatures were maintained for a longer period than for other yards.

Bacterial profiles were similar in Phase II for all compost yards despite variations in pasteurization time and practice (i.e., pasteurization in bulk or in trays). The common taxa across all yards were *Xanthomonodaceae* (mainly *Pseudoxanthomonas* (2–31%)) and *Streptosporagiaceae* (*Thermopolyspora* (1–2.5%)). In Yards B, C and E, a smaller proportion of *Pseudoxanthomonas* (2–10%) in the bacterial profile corresponded with a larger proportion of other thermophilic taxa such as *Thermobacillus* (1%), *Thermopolyspora* (2–4%) and *Truepera* (2–4%) (Table S3). Although the *Xanthomonadaceae* family was not dominant in Yard E (Fig. 4), *Pseudoxanthomonas* was one of the top 10 genera by the end of Phase II at this yard (Table S3).

### Bacterial diversity changes in a similar manner during composting at all yards

**Fig. 5:**
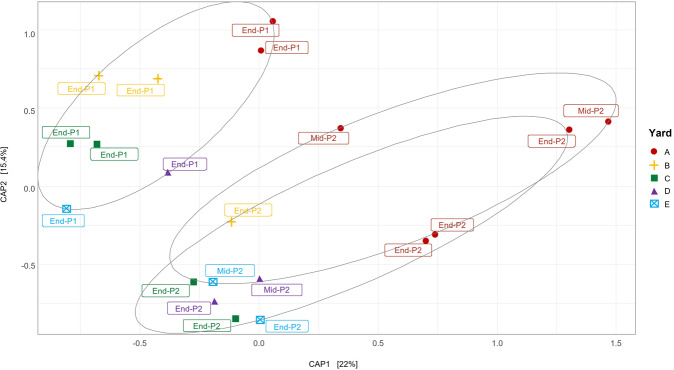
Canonical analysis of principal components of the bacterial communities from five geographically distinct compost yards. Unweighted Unifrac statistical analysis was used to use to measure the differences in bacterial communities in compost yards (CAP1) and phases (CAP2). Ellipses contain samples from end-Phase 1, mid-Phase 2, and end-Phase 3.

An unweighted Unifrac distance metric was used to compare bacterial communities among compost yards and phases (Fig. 5). The succession of different bacterial communities throughout the composting process followed the same general pattern in all compost yards. This was indicated with end-Phase I samples at the top left of the plot, progressing to end-Phase II at the bottom right (Fig. 5). The bacterial community at end-Phase I was significantly different from that in the mid- and end-Phase II (PERMANOVA: *F* = 2.4399, *R*^2^ = 0.1764, *p* < 0.05, d.f. = 3). The bacterial communities in Yard A clustered separately from other yards in the ordination plot, presumably due to the combination of large operational scale and indoor Phase I processing (Table S1).

## Discussion

Reproducible, commercial yields of button mushrooms can only be achieved if consistent compost quality is guaranteed. Because composting is a microbial process, we hypothesized that the microbial communities responsible for transformation of a uniform composting substrate (wheat straw/poultry manure) into productive compost will also show a degree of consistency. In this study, we examined fungal and bacterial communities present in the compost throughout the composting process (19 time points, from raw materials to commencement of cropping), to determine the succession of microbes present. Importantly, we also compared the compost bacterial communities in five geographically separate composting facilities across south-eastern Australia, to determine whether a consistent composting process is reflected in similar microbial communities. This study extends recent reports which have focussed on individual composting yards [16, 17, 19], and also builds on work done in previous decades, which compared mushroom yields and quality at a large number of facilities over multiple years [43–45].

The detailed timeline study revealed rapid succession of both bacterial and fungal taxa throughout Phase I (Fig. 3). The dominant bacterial taxa included soil and plant-related bacteria like *Acinetobacter* and *Bacillus*, *Arcobacter* (presumably derived from the poultry manure), and several other genera. Fungal diversity also varied, with *Lewia* dominating initially (probably derived from the wheat straw, as it is commonly associated with cereals [46]) followed by *Myceliophthora*, a cellulose-degrading genus that has also been found in *Agaricus subrufescens* compost [47, 48]. In Phase II, the fungal community was entirely dominated by *Mycothermus thermophilus*. The Phase II bacterial community contained high levels of *P. taiwanensis*, which was the dominant taxon not only in the detailed timeline study (Fig. 3), but also in three out of five compost yards tested for comparison (Fig. 4). However, several other taxa were also consistently present in Phase II compost from all compost yards studied, particularly *Chelativorans*, *Pseudoxanthomonas* and *Thermopolyspora. Pseudoxanthomonas taiwanensis* has been identified as a key species in other mushroom composts [19, 25], including oyster mushroom compost [49], and in other cellulose degrading consortia [50]. *Pseudoxanthomonas taiwanensis* is also dominant in the thermophilic stage of compost preparation for oyster mushrooms, equivalent to Phase I [49], while Actinobacteria, such as *Thermopolyspora*, and Bacilli, such as *Thermobacillus* and *Ureibacillus*, dominate mature oyster mushroom compost [49].

Much of the cellulose breakdown occurs during Phase II [3, 16] and the dominance of *Pseudoxanthomonas* at this time suggests that it might play a significant role by boosting cellulose degradation. *Pseudoxanthomonas taiwanensis* is known to promote cellulose degradation in consortia used in biofuel production [50–52] but, paradoxically, it does not degrade cellulose when in pure culture, and it does not appear to harbor genes encoding cellulase. It has been suggested that its influence in consortia is due either to its production of β-glucosidase [50] or its contribution to acetate removal and pH control [51], but experiments to confirm this have been inconclusive. The role played by *Pseudoxanthomonas* in cellulose breakdown must therefore be involved with the other microbes and further work is needed to explore this complex relationship.

Where *Pseudoxanthomonas* (2–4%) was not the dominant organism in end-Phase II samples, there was a higher proportion of Actinobacteria (8–12%) and Bacilli (1–1.5%) in the bacterial profile (Fig. 4). In Yards C and E, *Xanthomonadaceae* appeared in end-Phase I compost and the proportion was higher than in their respective end-Phase II samples (Fig. 4). The dominant actinomycete in Yards C and E was *Thermopolyspora* (Table S3). This pattern has been found in composts that use chemical N or other straw materials (e.g., alfalfa) as their main N source [19, 53]. For mushroom compost produced in China, for example, high throughput sequencing showed that *P. taiwanensis* was the dominant organism in mid- to end-Phase I samples and *Thermobispora*, an actinomycete, was the dominant organism in end-Phase II samples [19], with the proportion of Bacilli being significantly smaller compared to the actinomycete population [19]. Phase I compost for this study in China was done in windrows and details of the temperatures attained during the study were not provided [19], but it would seem likely that temperatures were substantially lower than the 80 °C reached in bunkers in Australian compost yards.

Actinobacteria and Bacilli are important in various composting systems [49, 54, 55]. *Thermobifida, Thermomonospora* and *Thermopolyspora* were among the most abundant of thermophilic Actinobacteria found both in this study and in other studies [19, 56, 57]. These genera are known for their cellulose degrading enzymes; *Thermopolyspora* and *Thermomonospora* produce hemicellulases [57–59] while *Thermobifida cellulolytica* is able to completely degrade cellulose [60]. *Thermopolyspora* dominated the actinomycete community of end-Phase II compost in this study and a similar result was found in end-Phase II compost derived from different straw types [56]. Actinobacteria and Bacilli also dominate in mature oyster mushroom compost [49]. *Geobacillus* and *Ureibacillus* have both been isolated from compost samples in this study (data not shown) as well as from other composts [61, 62]. These taxa are often found in cellulose degrading systems [63], particularly composts, due to their heat resistant, spore-forming nature and their highly active lignocellulolytic enzymes [64].

Another important genus in mushroom compost is the α-Proteobacterium *Chelatococcus* (family *Beijerinckiaceae*). In this study, *Chelatococcus daeguensis* was the most common species of this genus and was found in all yards sampled. *Chelatococcus daeguensis* is able to grow on several C sources, including cellobiose [65], and it has been proposed that *C*. *daeguensis* aids lignocellulose degradation by activating lignin breakdown [63].

From a technical point of view, the Phase I process was the most variable step among the compost yards studied. Phase I is a partially controlled process that takes place in an enclosed bunker or in ricked windrows. Compost temperatures are initially mesophilic (25–45 °C) and rise to thermophilic conditions (80 °C) [21, 66] as microbial activity increases, and is controlled by the air supply to the compost. Differences among facilities are likely to have occurred because older yards rely on mechanical turning of the compost for aeration, while newer compost yards provide additional aeration to the compost pile through an aerated floor (maintaining at least 5% oxygen concentration in the compost [21, 67, 68]). Phase I is complete when the ammonia concentration reaches levels of 150–800 ppm [69, 70], (due to proteolysis and ammonification) and the time required for this trigger ranged from 9–21 days in this study (Table S1). All these factors influence the succession of microorganisms that degrade the increasingly complex organic matter derived from raw material [17, 25, 26].

The variability in process in Phase II was much less than Phase I. Phase II composting is done in enclosed tunnels over 6 days, and temperature and oxygen supply are more closely controlled than during Phase I [71]. In contrast to Phase I composting, Phase II is initially thermophilic (60 °C) during pasteurization and decreases to mesophilic conditions (reduced from 55–25 °C) during conditioning. Following pasteurization, conditioning occurs with a slow decrease in temperature from 55–45 °C (then rapidly cooled to 25 °C for colonization by *A*. *bisporus*), which is the ideal temperature range for Actinobacteria and fungi to reassimilate free ammonia back into the compost [48, 69, 72]. Although all the yards studied showed very similar management of Phase II, the variation in composting scale (i.e., the size of the facility) and the peak temperatures achieved in Phase I also appear to be important in establishing the bacterial profile in Phase II.

When the abundance of *Pseudoxanthomonas* was low, the abundances of Actinobacteria and Bacilli were greater (Fig. 4). This was most likely due to the temperatures achieved and the process used in Phase I (Table S1). Phase I was done outdoors for Yards B–E and the temperature profiles were therefore more variable than the indoor process of Yard A (Table S1). However, due to the larger scale of Yard D compared to Yards B, C and E, larger compost piles may have been able to reach higher temperatures (Table S1). In studies of a range of composts that did not reach a peak of 80 °C (mushroom compost and partial green waste compost), the bacterial community favored more Actinomycetes, whereas when temperatures were greater than 80 °C, the bacterial profile had more Bacilli and *P. taiwanensis* [25, 54].

Although the dominant bacteria in the five compost yards sampled were clearly variable (Fig. 4), the overall bacterial communities for each phase clustered relatively closely together in the ordination plot (Fig. 5), suggesting a high degree of similarity. Bacterial diversity was clearly different between end-Phase I and Phase II, and all Phase I and Phase II communities followed the same pattern of change (Fig. 5). This suggests that despite the variability in Phase I composting, the Phase II composting process selects for bacteria that fulfill similar roles in transforming raw materials into the desired selective growth substrate for *A*. *bisporus*.

## Supplementary information

Supplementary data

Supplementary Tables 3-5

## Data Availability

Sequencing data are available at NCBI SRA under BioProject PRJNA867030.
