# Supplementary material for: Dynamics of microbial community and enzyme activities during preparation of Agaricus bisporus compost substrate
Source: ISME Commun. 2022 Sep 23;2:88. doi: 10.1038/s43705-022-00174-9 (PMC9723551; doi:10.1038/s43705-022-00174-9)
Supplement: Supplementary file 1 — Supplementary data [file 43705_2022_174_MOESM1_ESM.pdf]

Supplementary data

Dynamics of microbial community and enzyme activities during preparation of *Agaricus bisporus* compost substrate

Meghann Thai, Katarzyna Safianowicz, Tina L. Bell and Michael A. Kertesz*

School of Life and Environmental Sciences, The University of Sydney, Sydney, NSW, 2006, Australia.

*Corresponding author: Michael A. Kertesz

School of Life and Environmental Sciences,

The University of Sydney,

Sydney, NSW, 2006, Australia

Supplementary tables

Table S1. Variations in the composting process at five geographically distinct compost yards.

Table S2. Details for analytical methods used for measuring enzyme activity for compost samples.

Table S3, Table S4 and Table S5 are extensive data files and are presented in a separate supplementary Excel file.

Table S3. Bacterial diversity in compost at selected timepoints during composting. Values are relative percent.

Unassigned OTUs are not shown.

Table S4. Fungal diversity in compost at selected timepoints during composting.

Unassigned OTUs are not shown.

Table S5. Bacterial diversity in compost from five geographically distinct compost yards.

Unassigned ASVs are not shown.

Supplementary figures

Figure S1. Rarefaction analysis of bacterial and fungal sequences. A. Bacterial partial 16S sequences, experiment 1. B. Fungal ITS sequences, experiment 1. C. Bacterial partial 16S sequences, experiment 2.

Figure S2. Changes in pH and electrical conductivity during composting: (A) pH, (B) electrical conductivity.

Figure S3. Nutrient content of compost at selected times during composting: **(**A) soluble carbon, (B) soluble nitrogen, (C) total carbon, (D) total nitrogen, (E) carbon/nitrogen ratio.

Figure S4. Moisture levels in compost samples at different stages of composting.

Table S1. Variations in the composting process at various yards around Australia.

**Compost yard**	**Average rainfall (mm)**	**Average temperature (°C)**	**Yard size* (t per crop)**	**Raw materials**	**Prewet▲ (48 h-14 days)**	**Phase I• (9-21 days)**	**Phase II♦ (6-10 hrs)**
A	700	Min: 11.5<br>Max: 25.4	Large	Wheat straw<br>Poultry manure<br>Gypsum<br>Added nitrogen supplements	Medium duration<br>Aerated<br>Indoor	Medium duration<br>Aerated indoor bunker<br>Crop turned three times	Medium duration pasteurization<br>Bulk
B	400	Min: 8.7<br>Max: 22.1	Medium	Wheat straw<br>Poultry Manure<br>Gypsum	Medium duration<br>Aerated<br>Outdoor	Short duration<br>Aerated outdoor bunker<br>Crop turned two times	Long duration pasteurization<br>Bulk
C	390	Min: 11.5<br>Max: 23.5	Small	Wheat straw<br>Poultry manure<br>Gypsum<br>Added nitrogen supplements	Medium duration<br>Outdoor	Medium duration<br>Aerated outdoor bunker<br>Crop turned five times	Medium duration pasteurization<br>Bulk
D	480	Min: 10.1<br>Max: 21.3	Large	Wheat straw<br>Poultry manure<br>Gypsum	Short duration<br>Outdoor	Long duration<br>Outdoor concrete slab in windrows<br>Crop turned 10 times	Short duration pasteurization<br>Trays
E	830	Min: 7.7<br>Max: 16.9	Small	Wheat straw<br>Poultry manure<br>Gypsum<br>Added nitrogen supplements	Long duration<br>Outdoor	Medium duration<br>Outdoor concrete slab in windrows<br>Crop turned six times	Short duration pasteurization<br>Trays

*Yard size – Small: 80-200 tonnes (t) of Phase I compost, medium: 200-800 t of PI, large: >800 t of PI

▲Timing of pre-wet – Short: <3 days, medium: 3-6 days, long: >7 days

•Timing of Phase I – Short: <12 days, medium: 12-18 days, long: >19 days

♦Timing of holding period for Phase II – Short: <7 h, medium: 8-9 h, long: >10 h

Table S2. Details of enzyme analytical methods for compost samples.

Enzyme	Substrate	Temperature (°C)	Maximum reaction time (min)	Detection wavelength (nm)	Reference
Protease (alanine aminopeptidase)*	L-alanine-7-amido-4-methylcoumarin	37	180	350/460	(1)
Amylopectinase	Amylopectin	50	120	450	(2)
β-glucosidase*	4-MUF-β-D-glucoside	37	180	350/450	(1)
Cellulase	Low viscosity carboxymethyl-cellulose	50	120	450	(2, 3)
Chitinase*	β-1,4-N-acetylglucosaminide	37	180	350/450	(1)
Fluorescein diacetate hydrolysis*	Fluorescein diacetate	25	40	485/520	(4)
Invertase	Saccharose	50	120	450	(2, 3)
Peroxidase	Tetramethylbenzidine + hydrogen peroxide	25	1	450	(5)
Xylanase	Xylan	50	120	450	(2, 3)

* indicates fluorescent substrate. MUF – methylumbelliferone

References

1. Freeman C, Liska G, Ostle NJ, Jones SE, Lock MA. The use of fluorogenic substrates for measuring enzyme activity in peatlands. Plant Soil. 1995;175:147-52.
2. Jue CK, Lipke PN. Determination of reducing sugars in the nanomole range with tetrazolium blue. J Biochem Biophys Meth. 1985;11:109-15.
3. Schinner F, von Mersi W. Xylanase activity, CM-cellulase activity and invertase activity in soil - an improved method. Soil Biol Biochem. 1990;22:511-5.
4. Shaw LJ, Burns RG. Enzyme activity profiles and soil quality. In: Bloem J, Hopkins DW, Benedetti A, editors. Microbial methods for assessing soil quality. Wallingford: CABI Publishing; 2006. p. 158-82.
5. Johnsen AR, Jacobsen OS. A quick and sensitive method for the quantification of peroxidase activity of organic surface soil from forests. Soil Biol Biochem. 2008;40:814-21.

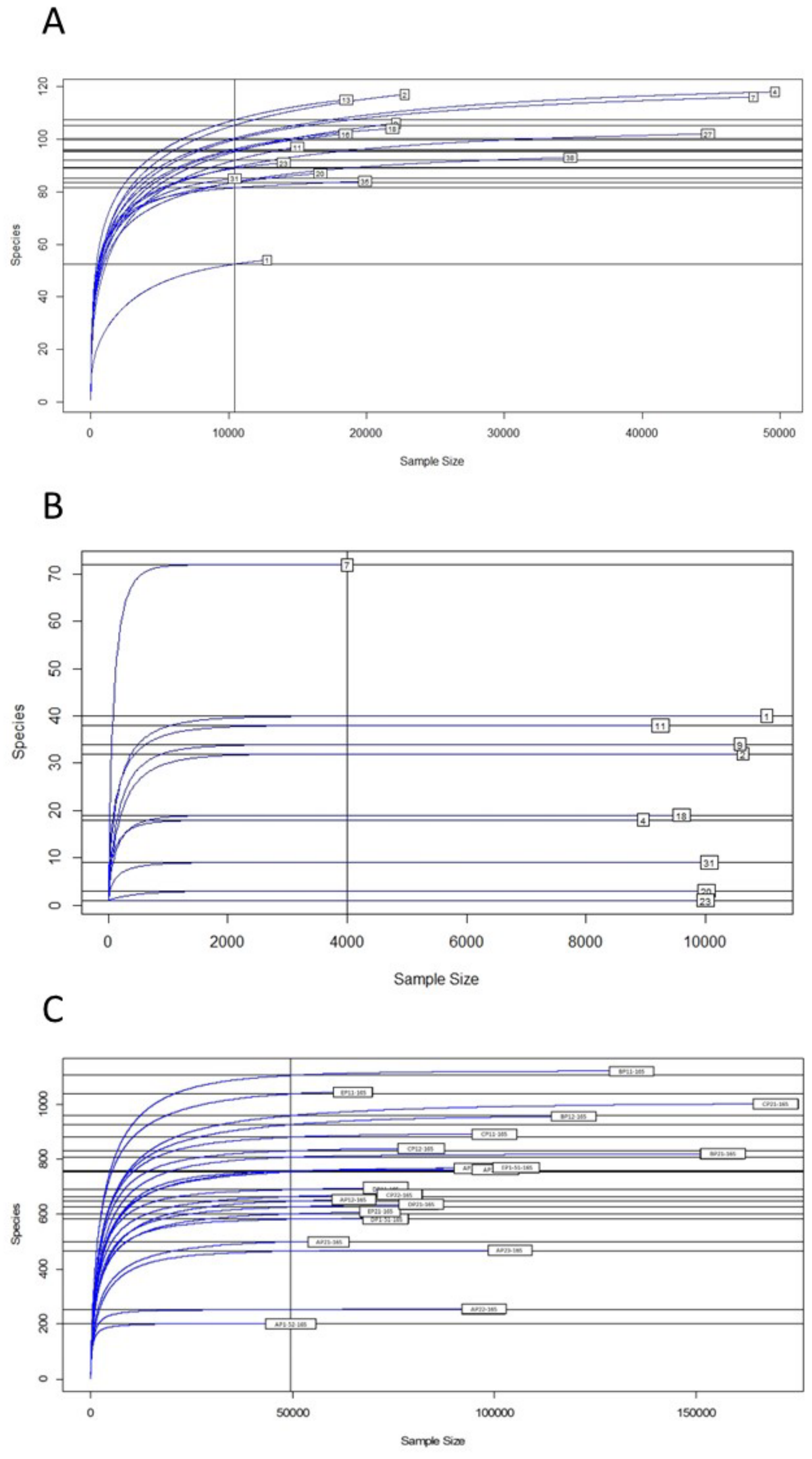

Figure S1. Rarefaction analysis of bacterial and fungal sequences. A. Bacterial partial 16S sequences, experiment 1. B. Fungal ITS sequences, experiment 1. C. Bacterial partial 16S sequences, experiment 2.

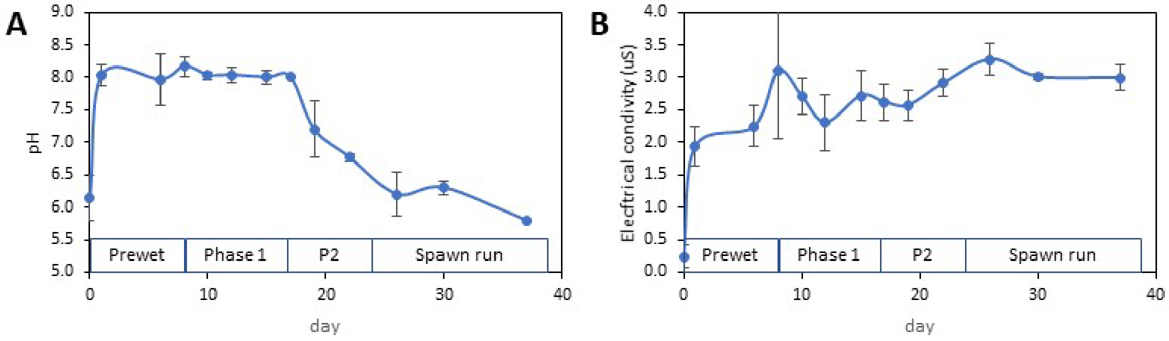

Figure S2. Changes in pH and electrical conductivity during composting: (A) pH, (B) electrical conductivity.

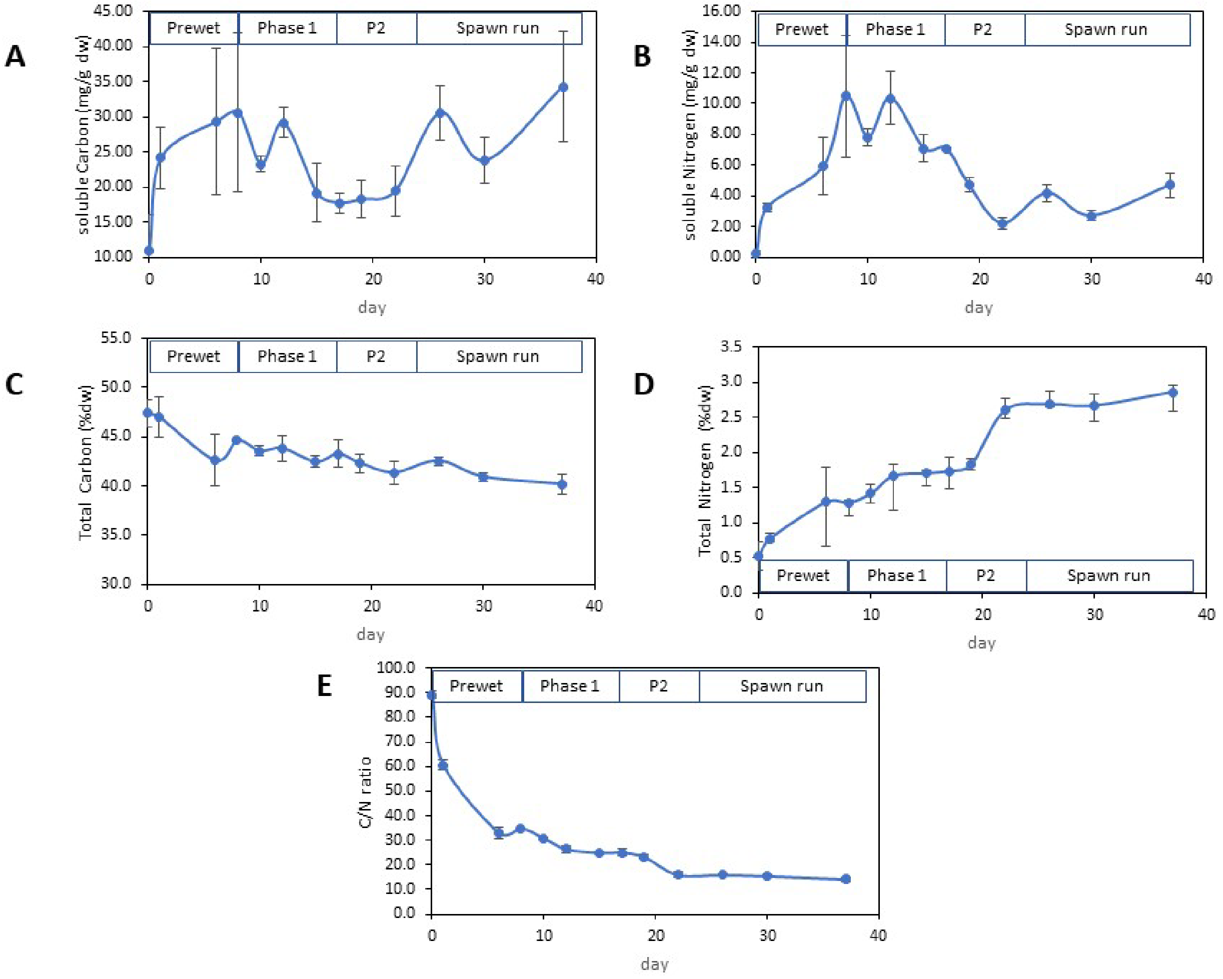

Figure S3. Nutrient content of compost at selected times during composting: **(**A) soluble carbon, (B) soluble nitrogen, (C) total carbon, (D) total nitrogen, (E) carbon/nitrogen ratio.

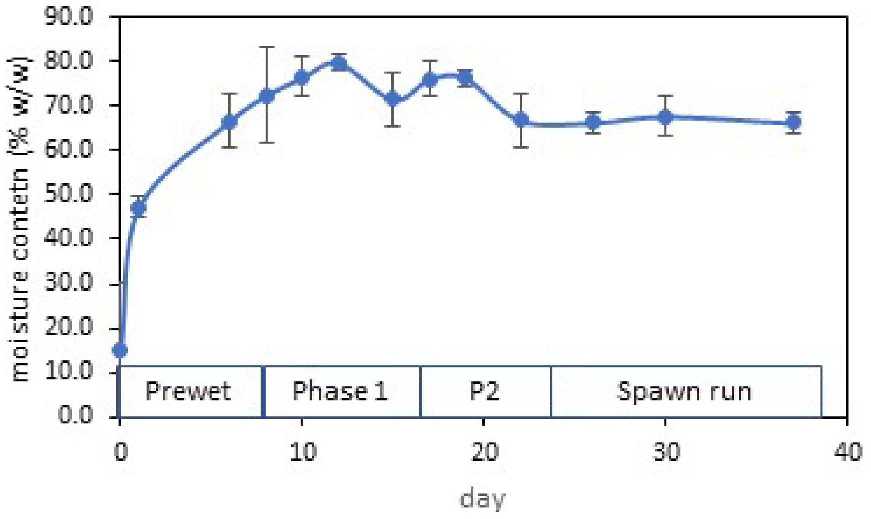

Figure S4. Moisture levels in compost samples at different stages of composting.
